# Prognostic and predictive values of tumour budding in stage IV colorectal cancer

**DOI:** 10.1002/bjs5.50300

**Published:** 2020-05-29

**Authors:** K. Nagata, E. Shinto, M. Yamadera, T. Shiraishi, Y. Kajiwara, K. Okamoto, S. Mochizuki, K. Hase, Y. Kishi, H. Ueno

**Affiliations:** ^1^ Department of Surgery National Defence Medical College 3‐2 Namiki Tokorozawa 359‐8513 Japan

## Abstract

**Background:**

Tumour budding is an important prognostic feature in early‐stage colorectal cancer, but its prognostic significance in metastatic disease has not been fully investigated.

**Methods:**

Patients with stage IV disease who had primary colorectal tumour resection without previous chemotherapy or radiotherapy from January 2000 to December 2018 were reviewed retrospectively. Budding was evaluated at the primary site and graded according to the criteria of the International Tumor Budding Consensus Conference (ITBCC) (BD1, low; BD2, intermediate; BD3, high). Patients were categorized by metastatic (M1a, M1b) and resectional (R0/R1, R2/unresected) status. Subgroups were compared for overall (OS) and recurrence‐free (RFS) survival in R0/R1 subgroups; R2/unresected patients were evaluated for the rate of tumour progression, based on change in tumour size from baseline.

**Results:**

Of 371 patients observed during the study, 362 were analysed. Patients with BD3 had a lower 5‐year OS rate than those with BD1 + BD2 (18·4 *versus* 40·5 per cent; *P* < 0·001). Survival analyses according to metastatic and resection status also showed that BD3 was associated with shorter OS than BD1 + BD2. In multivariable analysis, BD3 (hazard ratio (HR) 1·51, 95 per cent c.i. 1·11 to 2·10; *P* = 0·009), T4 status (HR 1·39) and R2/unresected status (HR 3·50) were associated with decreased OS. In the R0/R1 subgroup, the 2‐year RFS rate was similar for BD3 and BD1 + BD2 according to metastatic status. There was no significant difference between BD3 and BD1 + BD2 for change in tumour size in the R2/unresected subgroup (*P* = 0·094). Of 141 patients with initially unresectable metastases who had chemotherapy, 35 achieved conversion from unresectable to resectable status. The conversion rate was significantly higher for BD1 + BD2 than for BD3 (36 *versus* 18 per cent; *P* = 0·016).

**Conclusion:**

Stage IV colorectal cancer with high‐grade tumour budding according to ITBCC criteria correlates with poor prognosis.

## Graphical abstract

In stage IV colorectal cancer, tumour budding graded by International Tumor Budding Consensus Conference criteria is an important morphological marker that can predict prognosis and, possibly, tumour chemoresistance. The clinical relevance was maintained consistently in both resection subgroups (R0/R1 and R2/unresected).

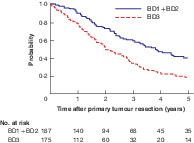

Tumor budding and colorectal metastasis

## Abstract (es)

**Antecedentes:**

La esofaguectomía por cáncer se asocia con un descenso de la calidad de vida relacionada con la salud (*health‐related quality of life*, HRQoL) a largo plazo. El objetivo de este estudio fue evaluar el efecto de las comorbilidades sobre la HRQOL entre pacientes supervivientes de cánceres de esófago o de la unión gastroesofágicas después de 10 años o más.

**Métodos:**

Este estudio incluye una cohorte de base poblacional recogida de forma prospectiva que incluía todos los pacientes operados de cáncer de esófago o de la unión gastroesofágica en Suecia en 2001‐2005 con seguimiento hasta el 31 de diciembre de 2016. Todos los datos relacionados con las características de los pacientes y del tumor, detalles del tratamiento y HRQoL se recogieron en una base de datos prospectiva. Se utilizaron modelos de regresión multivariable ANCOVA, ajustados por edad, sexo, histología del tumor, estadio, y técnica quirúrgica, para calcular las puntuaciones medias ajustadas con los i.c. del 95% para todas las variables de la HRQoL.

**Resultados:**

Un total de 92 (88%) supervivientes respondieron a los cuestionarios. En función del impacto de las comorbilidades en la salud en general, se clasificaron a los pacientes en los grupos de bajo *versus* alto impacto. Los resultados muestran que los pacientes en el grupo de alto impacto presentaban un descenso clínicamente significativo de la HRQoL y un aumento en el nivel de síntomas, pero las diferencias entre estos dos grupos no fueron estadísticamente significativas.

**Conclusión:**

A los 10 años de la esofaguectomía por cáncer, las comorbilidades con un alto impacto sobre la salud general siguen contribuyendo en el deterioro de la HRQoL.

## Introduction

In 2018 more than 1·8 million people were diagnosed with colorectal cancer and 880 000 died from the disease worldwide^1^. Although recent advances in medical screening have provided considerable opportunity for detecting early‐stage colorectal cancer, approximately 20 per cent of patients have distant metastases at the time of presentation^2^, ^3^. For patients with synchronous distant metastases, median overall survival (OS) is reportedly about 30 months and the 5‐year OS rate has been estimated at around 12 per cent^4^. However, the prognosis of patients with stage IV colorectal cancer correlates with the resectability of metastatic lesions, and prognostic analyses have therefore been performed categorizing patients who have a curative resection and those who do not.

Tumour budding is a histological feature observed predominantly at the tumour front. Previous studies have revealed the clinical significance of budding, not only as a predictor of recurrence in stage II disease^5^, ^6^, but also as a predictor of recurrence and chemosensitivity in stage III colorectal cancer^7^, ^8^, ^9^, ^10^, ^11^, ^12^. However, in patients with stage IV disease, the significance of tumour budding remains unclear, because most analyses of stage IV colorectal cancer have had limited patient numbers^11^, ^12^, ^13^.

This study aimed to investigate the prognostic impact of tumour budding in patients with stage IV CRC.

## Methods

All consecutive patients diagnosed with stage IV colorectal cancer who did not undergo chemotherapy or radiotherapy and had a primary tumour resection between January 2000 and December 2018 at the National Defence Medical College Hospital, a general hospital affiliated to the medical college in Japan, were reviewed retrospectively.

Tumour stages were categorized according to the 7th edition of the UICC TNM classification. Patients with peritoneal metastasis in a limited area near the original tumour, resectable para‐aortic lymph node metastasis and a single hepatic metastasis near the liver surface underwent synchronous metastatic resection in addition to primary colorectal cancer resection.

According to the departmental protocol, patients had a metachronous resection with no chemotherapy if they presented with fewer than five hepatic metastatic lesions smaller than 5 cm. However, patients with larger liver metastases and those with more than five liver metastases were treated with chemotherapy, followed by surgical resection if considered suitable. All patients with resectable lung metastasis were resected after chemotherapy. Patients with DNA mismatch repair deficiency by examination for MutL homologue 1 (MLH1) or MutS homologue 2 (MSH2) immunohistochemistry were excluded because these mutations are associated with a chemotherapy‐resistant property.

The study was approved by the ethics committee of the National Defence Medical College Hospital. Written informed consent was obtained from each patient in accordance with institutional regulations.

Patients were categorized according to tumour metastatic status (M1a, metastases in a single organ; M1b, metastases in the peritoneum or multiple organs). In addition, patients were categorized according to type of treatment for metastatic sites, defined as: R0, complete resection (no residual tumour); R1, macroscopically complete resection (microscopic residual tumour); R2, macroscopically incomplete resection (macroscopic residual tumour); and unresected, unresected metastatic lesions (surgery for metastatic sites could not be done).

Data regarding patient and treatment‐related characteristics, including age at time of surgery, sex, resection status and chemotherapy, were extracted from electronic patient records. The following tumour characteristics were also recorded: tumour location, depth of tumour invasion, histological type, venous invasion, lymphatic invasion, tumour budding, node metastasis and distant metastasis. In R2/unresected patients undergoing chemotherapy for metastasis, the response of the tumour was evaluated and categorized as described below.

### Immunohistochemistry

Immunohistochemical staining of MLH1 (clone G168‐15; BD Biosciences, San Jose, California, USA) and MSH2 (FE11; Invitrogen, Carlsbad, California, USA) were used to verify retrospectively the microsatellite instability status, as reported previously^14^. Tumour cells were judged to be negative for protein expression only when they lacked staining in a sample in which healthy colonocytes and stroma cells were stained. The normal colonic crypt epithelium adjoining the tumour was used as an internal control. Both MLH1 and MSH2 proteins should stain positively in nuclei when they are expressed^15^. Cancers with negative MLH1 or MSH2 expression were considered to have DNA mismatch repair deficiency.

### Tumour budding

Tumour budding was scored according to the International Tumor Budding Consensus Conference recommendations^16^. Haematoxylin and eosin‐stained sections were scanned at medium power to identify the densest area of budding at the tumour front (the ‘hotspot within a field measuring 0·785 mm^2^’^16^). Tumour buds were counted in this area with a × 20 objective lens. The final bud count and the budding category (BD1, 0–4 buds; BD2, 5–9 buds; BD3, 10 or more buds) were recorded. Budding was categorized into two grades: BD1 + BD2 and BD3, as described previously^14^, ^15^.

### Outcome measures

Outcome measures included 5‐year OS (defined as the time from the date of primary tumour resection to death from any cause) and recurrence‐free survival (RFS) (defined as the time from the date of complete macroscopic resection to radiographic detection of recurrence in patients with an R0/R1 resection). In R2/unresected patients undergoing chemotherapy for metastasis, the change in tumour size (CTS) from baseline was calculated.

When chemotherapy led to a reduction in tumour size, the CTS was defined as maximal tumour shrinkage observed in a patient (percentage tumour shrinkage, based on the longest diameter, observed at the lowest point compared with baseline, similar to the depth of response)^17^, estimated from the smallest size of tumour after chemotherapy. In patients with continuous tumour progression despite chemotherapy, the CTS was defined as the percentage of tumour enlargement 3 months after chemotherapy, estimated by dividing the size of the growing tumour (3 months after chemotherapy) by the baseline tumour size. Of note, the timing of CTS assessment (for tumour shrinkage) varies between patients, and maximum tumour shrinkage usually occurs 3–6 months after the start of first‐line therapy^18^. Among the latter subgroup of patients, disease progression was defined as a 20 per cent increase in tumour diameter 3 months after chemotherapy, compared with the baseline (CTS greater than 120 per cent).

In addition, in patients with initially unresectable metastases undergoing chemotherapy, the conversion rate according to BD category was explored.

### Follow‐up

Patients with colorectal cancer were followed up routinely at the outpatient clinic. Follow‐up included physical examination, tumour marker evaluation, follow‐up imaging (primarily contrast‐enhanced CT) and colonoscopy according to the Japanese Society for Cancer of the Colon and Rectum guidelines^19^, with modifications based on patient need.

### Statistical analysis

Groups were compared with the χ^2^ or Fisher's exact test, with comparison of age values by Student's *t* test. OS and RFS were estimated using the Kaplan–Meier method and compared with the log rank test. The Cox proportional hazards model was employed for multivariable analysis; only factors with log rank *P* < 0·050 were included in the regression model. Patient age, sex, tumour location, depth of tumour invasion, histological type, venous/lymphatic invasion, tumour budding, node metastasis, metastatic status, resection status and chemotherapy were used as co‐variables. All statistical analyses were obtained using JMP® 13 software (SAS Institute, Cary, North Carolina, USA). *P* < 0·050 was considered statistically significant.

## Results

**Figure 1:**
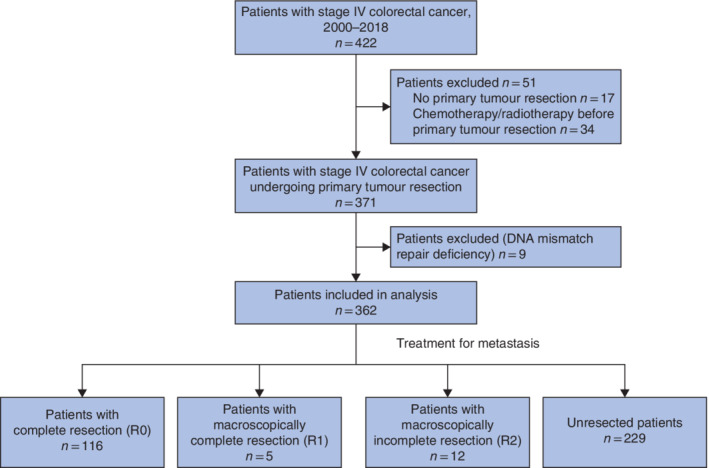
Flow diagram for the study

Some 371 patients were diagnosed and treated for stage IV colorectal cancer in the study period, but nine were excluded because of their DNA mismatch repair deficiency status, leaving 362 patients for data analysis (*Fig*. 1). The median age was 67 (range 28–91) years, the M : F ratio was 223 : 139, and 27·3 per cent of patients had a right‐sided tumour (*Table* 1).

**Table 1:** Clinicopathological features in patients with stage IV colorectal cancer

		Tumour budding grade			Metastatic status		
	Total (*n* = 362)	BD1 + BD2 (*n* = 187)	BD3 (*n* = 175)	*P* ¶	M1a (*n* = 204)	M1b (*n* = 158)	*P* ¶
**Age (years)** *	67 (28–91)	67 (28–91)	66 (28–87)	0·403#	66 (28–88)	67 (32–91)	0·619#
**Sex ratio (M** : **F)**	223 : 139	112 : 75	111 : 64	0·489	131 : 73	92 : 66	0·246
**Tumour location**				< 0·001			0·107
Right side	99 (27·3)	31 (16·6)	68 (38·9)		49 (24·0)	50 (31·6)	
Left side	263 (72·7)	156 (83·4)	107 (61·1)		155 (76·0)	108 (68·4)	
**Depth of tumour invasion**				< 0·001			< 0·001
T1–3	160 (44·2)	102 (54·5)	58 (33·1)		109 (53·4)	51 (32·3)	
T4	202 (55·8)	85 (45·5)	117 (66·9)		95 (46·6)	107 (67·7)	
**Histological type**				0·041			0·640
Well/moderate†	308 (85·1)	166 (88·8)	142 (81·1)		172 (84·3)	136 (86·1)	
Poor/mucinous‡	54 (14·9)	21 (11·2)	33 (18·9)		32 (15·7)	22 (13·9)	
**Venous invasion**				0·026			0·005
Low	98 (27·1)	60 (32·1)	38 (21·7)		67 (32·8)	31 (19·6)	
High	264 (72·9)	127 (67·9)	137 (78·3)		137 (67·2)	127 (80·4)	
**Lymphatic invasion**				< 0·001			0·125
Low	238 (65·7)	148 (79·1)	90 (51·4)		141 (69·1)	97 (61·4)	
High	124 (34·3)	39 (20·9)	85 (48·6)		63 (30·9)	61 (38·6)	
**Tumour budding** §							0·007
BD1 + BD2	187 (51·7)	–	–		118 (57·8)	69 (43·7)	
BD3	175 (48·3)	–	–		86 (42·2)	89 (56·3)	
**Node metastasis**				< 0·001			0·121
Positive	299 (82·6)	140 (74·9)	159 (90·9)		163 (79·9)	136 (86·1)	
Negative	63 (17·4)	47 (25·1)	16 (9·1)		41 (20·1)	22 (13·9)	
**Metastasis**				0·007			
M1a	204 (56·4)	118 (63·1)	86 (49·1)		–	–	
M1b	158 (43·6)	69 (36·9)	89 (50·9)		–	–	
**Resection**				< 0·001			< 0·001
R0/R1	121 (33·4)	79 (42·2)	42 (24·0)		97 (47·5)	24 (15·2)	
R2	241 (66·6)	108 (57·8)	133 (76·0)		107 (52·5)	134 (84·8)	0·498
**Chemotherapy**				0·753			
Yes	285 (78·7)	146 (78·1)	139 (79·4)		158 (77·7)	127 (80·4)	
No	77 (21·3)	41 (21·9)	36 (20·6)		46 (22·5)	31 (19·6)	

Values in parentheses are percentages unless indicated otherwise;

* values are median (range).

† Well to moderately differentiated tubular adenocarcinoma;

‡ poorly differentiated adenocarcinoma or mucinous carcinoma;

§ grade of tumour budding: BD1, zero to four buds; BD2, five to nine buds; BD3, ten or more buds (per ×200 microscopic field).

¶ χ^2^ or Fisher's exact test, except #Student's *t* test.

Some 204 patients (56·4 per cent) had M1a disease, and the majority of metastases were located in the liver (161, 78·9 per cent), followed by lung (24, 11·8 per cent), lymph nodes (16, 7·8 per cent) and other (3, 1·5 per cent). Some 97 (47·5 per cent) of these 204 patients were treated with a complete/nearly complete resection (R0/R1), whereas 107 (52·5 per cent) had residual disease (R2/unresected). Metastases to two or more organs and/or the peritoneal surface (M1b status) were observed in 158 patients (43·6 per cent), of whom 24 (15·2 per cent) had a R0/R1 resection and 134 (84·4 per cent) had residual disease.

Thus, 121 patients were defined as having an R0/R1 resection. Among them, 33 had simultaneous resection of the primary tumour and metastases, whereas 88 were treated with metachronous procedures (median number of resections 1 (range 1–3)). In the R0/R1 subgroup, 87 patients had chemotherapy before metastatic resection, including 46 patients who had metachronous procedures aiming to reduce the size of metastatic lesions.

Among the 241 patients with R2/unresected status, 189 received chemotherapy, including oxaliplatin‐based chemotherapy (113 patients) and 5‐fluorouracil‐based chemotherapy without oxaliplatin (76).

Overall, tumour budding was classified as BD1 + BD2 in 187 patients (51·7 per cent) and as BD3 in 175 (48·3 per cent).

### Tumour budding and overall survival

**Figure 2:**
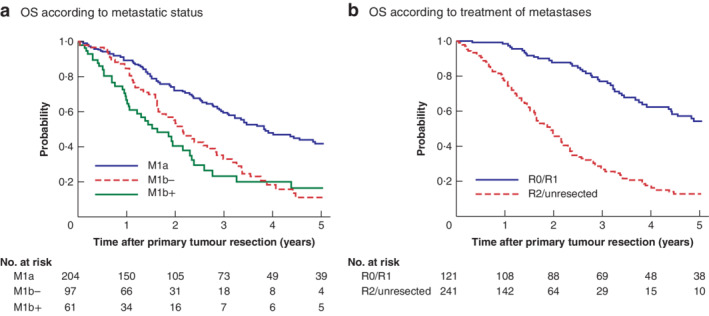
Kaplan–Meier analysis of overall survival in patients with stage IV colorectal cancer according to metastatic and metastasectomy status Overall survival (OS) according to **a** metastatic status and **b** treatment of metastases. M1b−, M1b status without peritoneal metastases; M1b+, M1b status with peritoneal metastases.

**Figure 3:**
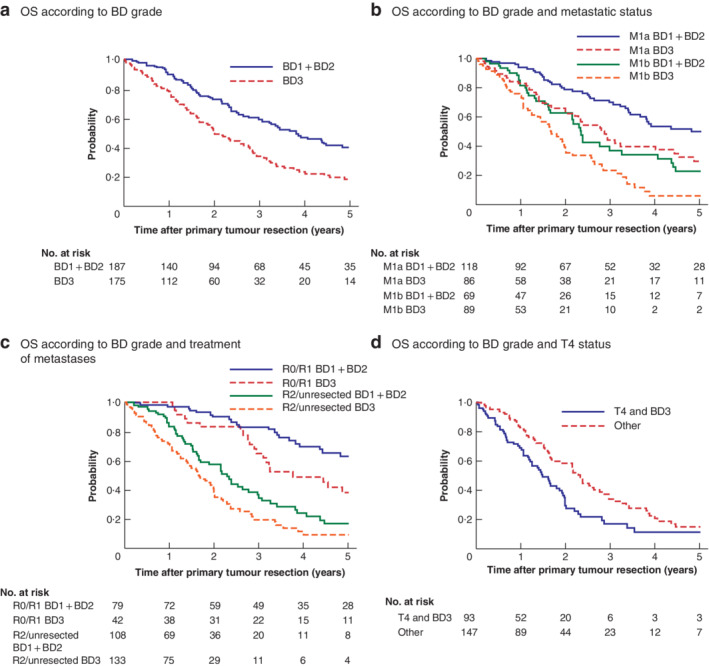
Kaplan–Meier analysis of overall survival in patients with stage IV colorectal cancer according to tumour budding, metastatic and metastasectomy status Overall survival (OS) according to **a** tumour budding (BD) grade of the primary lesion, **b** BD grade and metastatic status, **c** BD grade and treatment of metastases, and **d** BD grade and depth of tumour invasion (T4 category) of the primary lesion.

The median follow‐up was 19·9 (range 1·4–190·3) months. OS did not differ significantly between patients with M1b status with and without peritoneal metastases (5‐year OS rate: 14 *versus* 9 per cent respectively; *P* = 0·132) (*Fig*. 2 *a*). In addition, the 5‐year OS rate in the R0/R1 group was significantly higher than that in the R2/unresected group (54·3 *versus* 12·7 per cent respectively; *P* < 0·001) (*Fig*. 2 *b*). Patients with grade BD3 had a reduced OS compared with those with grade BD1 + BD2 (5‐year OS rate: 18·4 *versus* 40·5 per cent respectively, *P* < 0·001) (*Fig*. 3 *a*). Survival analyses according to metastatic status also showed that patients with BD3 had a shorter 5‐year OS rate than those with BD1 + BD2 (M1a status: 30 *versus* 50·2 per cent, *P* = 0·005; M1b status: 6 *versus* 23 per cent, *P* = 0·006) (*Fig*. 3 *b*). The same trend was documented for the R1/R0 subcategory (5‐year OS rate: 38 *versus* 63 per cent, *P* = 0·028) and the R2/unresected subcategory (5‐year OS rate: 9·3 *versus* 16·9 per cent, *P* = 0·007) (*Fig*. 3 *c*).

In univariable analysis for OS, depth of tumour invasion, node metastasis, tumour budding, metastasis status and resection status were significantly associated with OS. However, only depth of tumour invasion (hazard ratio (HR) 1·39, 95 per cent c.i. 1·02 to 1·92; *P* = 0·036), tumour budding (HR 1·51, 1·11 to 2·10; *P* = 0·009) and resection status (HR 3·50, 2·38 to 5·14; *P* < 0·001) retained prognostic value in the multivariable model (*Table* 2). In the R0/R1 subgroup (121 patients), tumour budding (HR 1·94, 1·08 to 3·43; *P* = 0·026) and metastasis status (HR 2·54, 1·25 to 4·83; *P* = 0·012) were independent prognostic factors in multivariable analysis (*Table* 3). In the R2/unresected subgroup (241 patients), depth of tumour invasion (HR 1·70, 1·08 to 2·51; *P* = 0·004) and tumour budding (HR 1·47, 1·04 to 2·09; *P* = 0·028) were independent prognostic factors in multivariable analysis. Survival curves combining these factors showed OS rates in the T4 and BD3 group to be lower than those in the other groups (3‐year OS rate: 17 *versus* 34·1 per cent respectively; 5‐year OS rate: 11 *versus* 14·7 per cent; *P* < 0·001) (*Fig*. 3 *d*). In particular, the strong association of BD3 status with poor OS was reported in the multivariable models regardless of resection status.

**Table 2:** Significance of clinicopathological parameters for overall survival

	Comparison of overall survival*		Multivariable analysis†	
	5‐year rate (%)	*P*	Hazard ratio	*P*
Age (≥ 70 *versus* < 70 years)	22·5 *versus* 32·8	0·111		
Sex (M *versus* F)	25·9 *versus* 39·3	0·549		
Tumour location (right *versus* left)	30·5 *versus* 30·2	0·290		
Depth of tumour invasion (T4 *versus* T1–3)	22·6 *versus* 40·6	< 0·001	1·39 (1·02, 1·92)	0·036
Histological type (well/moderate *versus* poor/mucinous)	30·4 *versus* 24·6	0·107		
Venous invasion (low *versus* high)	37·9 *versus* 27·4	0·123		
Lymphatic invasion (low *versus* high)	30·7 *versus* 29·3	0·182		
Tumour budding (BD3 *versus* BD1 + BD2)	18·4 *versus* 40·5	< 0·001	1·51 (1·11, 2·10)	0·009
Node metastasis (positive *versus* negative)	27·8 *versus* 40·7	0·033	1·08 (0·72, 1·65)	0·723
Metastasis (M1a *versus* M1b)	42·1 *versus* 12·2	< 0·001	1·30 (0·94, 1·80)	0·111
R0 resection (R0/R1 *versus* R2)	54·3 *versus* 12·7	< 0·001	3·50 (2·38, 5·14)	< 0·001
Chemotherapy (yes *versus* no)	29·3 *versus* 39·6	0·712		

Values in parentheses are 95 per cent confidence intervals.

* Kaplan–Meier analysis with log rank test;

† Cox proportional hazards model.

**Table 3:** Significance of clinicopathological parameters for overall survival according to R status

	Comparison of disease‐specific survival*		Multivariable analysis†	
	5‐year rate (%)	*P*	Hazard ratio	*P*
**R0/R1 resection (*n* = 121)**				
Age (≥ 70 *versus* < 70 years)	34.8 *versus* 61.3	0.057		
Sex (M *versus* F)	50.9 *versus* 60.8	0.474		
Tumour location (right *versus* left)	61.0 *versus* 52.3	0.223		
Depth of tumour invasion (T4 *versus* T1–3)	51.9 *versus* 56.3	0.493		
Histological type (well/moderate *versus* poor/mucinous)	54.7 *versus* 52.1	0.823		
Venous invasion (low *versus* high)	54.8 *versus* 54.2	0.583		
Lymphatic invasion (low *versus* high)	53.8 *versus* 54.2	0.862		
Tumour budding (BD3 *versus* BD1+BD2)	38.4 *versus* 63.4	0.028	1.94 (1.08, 3.43)	0.026
Node metastasis (positive *versus* negative)	53.4 *versus* 57.0	0.492		
Metastasis (M1a *versus* M1b)	59.4 *versus* 30.1	0.007	2.54 (1.25, 4.83)	0.012
Chemotherapy (yes *versus* no)	51.5 *versus* 68.8	0.288		
**R2 resection (*n* = 241)**				
Age (≥ 70 *versus* < 70 years)	10.6 *versus* 11.7	0.481		
Sex (M *versus* F)	8.6 *versus* 19.2	0.901		
Tumour location (right *versus* left)	9.8 *versus* 12.4	0.076		
Depth of tumour invasion (T4 *versus* T1–3)	8.8 *versus* 21.2	0.003	1.70 (1.18, 2.51)	0.004
Histological type (well/moderate *versus* poor/mucinous)	11.3 *versus* 10.2	0.266		
Venous invasion (low *versus* high)	22.5 *versus* 7.4	0.312		
Lymphatic invasion (low *versus* high)	11.1 *versus* 11.9	0.827		
Tumour budding (BD3 *versus* BD1+BD2)	9.3 *versus* 16.9	0.007	1.47 (1.04, 2.09)	0.028
Node metastasis (positive *versus* negative)	12.2 *versus* 8.3	0.609		
Metastasis (M1a *versus* M1b)	16.9 *versus* 8.6	0.377		
Chemotherapy (yes *versus* no)	13.9 *versus* 0.0	0.003	0.46 (0.29, 0.75)	0.002

* Kaplan–Meier analysis with log rank test;

† Cox proportional hazards model.

### Tumour budding and recurrence‐free survival

**Figure 4:**
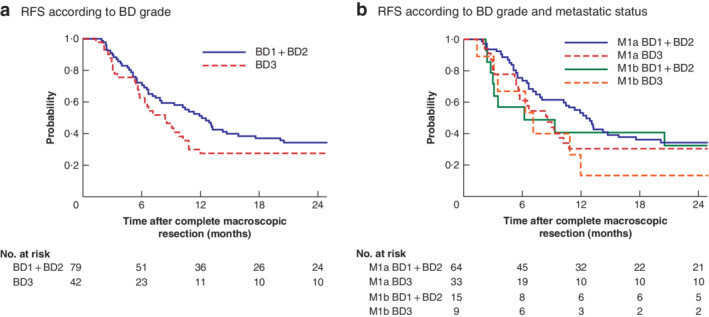
Kaplan–Meier analysis of recurrence‐free survival in R0/R1 resected patients according to tumour budding and metastatic status Recurrence‐free survival (RFS) according to **a** tumour budding (BD) grade in the primary lesion and **b** BD grade and metastatic status.

RFS was compared between BD3 and BD1 + BD2 groups in patients with R0/R1 resection status; RFS did not differ significantly (2‐year RFS rate: 24 *versus* 32 per cent respectively, HR 1·53 (95 per cent c.i. 0·98 to 2·34), *P* = 0·060) (*Fig*. 4 *a*). In addition, RFS analysis found no significant difference in the 2‐year RFS rate between BD3 and BD1 + BD2 groups associated with metastatic status (M1a: 27 *versus* 34 per cent, HR 1·48 (0·90 to 2·40), *P* = 0·112; M1b: 13 *versus* 33 per cent, HR 1·54 (0·57 to 4·08), *P* = 0·388) (*Fig*. 4 *b*).

### Tumour budding and change in tumour size

**Figure 5:**
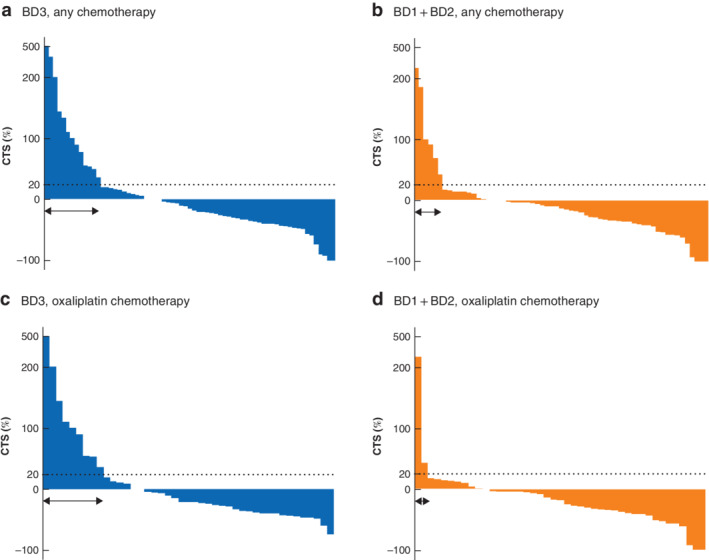
Waterfall plot of change in tumour size in individual patients who had chemotherapy after palliative primary resection Change in tumour size (CTS) from baseline in patients with tumour budding (BD) grade 3 (**a,c**) and BD1 + BD2 status (**b,d**) who had chemotherapy of any kind (**a,b**) or chemotherapy including oxaliplatin (**c,d**) after palliative primary resection. The black dotted line indicates a 20 per cent increase in longest tumour diameter. The arrows denote patients with a 20 per cent or greater increase in tumour diameter.

There was no significant difference in disease progression rate between BD3 and BD1 + BD2 groups (CTS: 20 *versus* 10 per cent respectively; *P* = 0·094) (*Fig*. 5 *a,b*). Among patients treated with oxaliplatin, the disease progression rate in the BD3 group was markedly higher than that in the BD1 + BD2 group (21·7 *versus* 4·7 per cent; *P* = 0·016) (*Fig*. 5 *c,d*). In addition, the 2‐year OS rate was lower in the BD3 group than in the BD1 + BD2 group among R2/unresected patients who had received chemotherapy (40·4 *versus* 57·3 per cent respectively; *P* = 0·042).

### Conversion rate in patients with initially unresectable metastases

Systemic chemotherapy was administered to 141 patients with initially unresectable metastases following primary tumour resection. Of these, 35 achieved conversion from an unresectable to a resectable status. The conversion rate was significantly higher in the BD1 + BD2 than in the BD3 group (36 *versus* 18 per cent; *P* = 0·016).

## Discussion

Several studies^20^, ^21^, ^22^, ^23^, ^24^, ^25^, ^26^, ^27^, ^28^ have reported that the TNM classification has significant prognostic value for stage IV colorectal cancer. Specifically, the type, number and spread of metastatic organs affect the prognosis of patients with stage IV disease. However, some researchers^26^, ^27^ have reported that pathological findings associated with biological attributes, such as differentiation grade and presence of vessel invasion, may be prognostic factors. With regard to the significance of tumour budding, the BD status in the primary lesion was reportedly an independent factor defining subsequent recurrence in patients with R0 resection of liver metastases^29^, ^30^; however, literature investigating tumour budding in patients with unresectable stage IV cancer is scant. In addition, there have been studies^11^, ^12^, ^13^ of budding that included patients with stage IV disease, but, although the positive prognostic significance of BD status was identified, failed to define robustly the true implications of BD status in patients with stage IV tumours because the number of such patients was too small.

In the present study, patients with BD3 status had a significantly shorter OS than those with BD1 + BD2 status, in both resection subcategories. Based on multivariable analysis, BD3, as well as R2/unresected status and T4 category, were documented to be independent poor prognostic factors, in line with the importance of the BD grade of the primary tumour as a prognostic factor in stage IV cancers.

Previous pathological studies^31^, ^32^, ^33^ have shown that BD is a morphological phenotype related to epithelial–mesenchymal transition (EMT)/partial EMT. EMT is a type of epithelial plasticity characterized by long‐lasting morphological and molecular changes in epithelial cells as a result of transdifferentiation towards a mesenchymal cell type. Cells that undergo EMT exhibit vigorous invasion and metastasis as well as chemoresistance^34^, ^35^. The aggressiveness underlain by the EMT may affect the early onset of the recurrence after curative resection. Furthermore, chemotherapeutic agents are widely administered in patients with R0/R1 resection as adjuvant treatment after complete macroscopic resection of distant metastasis or as systemic therapy after relapse. It is conceivable that the chemoresistant property of cancer might negatively affect disease control in all phases of anticancer treatment. The authors therefore believe that the molecular background of EMT has considerable influence on poor patient prognosis.

In R2/unresected patients without complete macroscopic surgical resection of metastases, the BD status and T category were independent poor prognostic factors. In this patient group, chemotherapeutic agents play a very important role during cancer therapy, and progression during treatment has a crucial negative prognostic impact. In accordance with the relationship between BD status and a possible favourable response to chemotherapy, the conversion rate in the BD1 + BD2 group was higher than that in the BD3 group (36 *versus* 18 per cent respectively). Consequently, relatively more patients with BD1 + BD2 status qualified for R0/1 resection among the initially unresectable cases, which resulted in a decline in the number of patients with BD1 + BD2 status in the R2/unresected group, rather than a decline in the number with BD3 status.

This study has some potential limitations. First, as a single‐centre retrospective study designed to examine prognostic factors related to long‐term OS, the case series included patients treated in 2000. In recent years, intensive systematic chemotherapy has occasionally been given before primary resection in line with the concept of metastatic lesion‐first. However, histopathological findings are changed when systematic chemotherapy is performed before resection of the primary tumour, and it may conceivably become difficult to evaluate the BD status correctly. Future studies will need to investigate the significance of BD grade of the primary tumour after neoadjuvant therapy to check the consistency of the usefulness of BD status.

## Disclosure

The authors declare no conflict of interest.
